# Genomic Characterization of a New Biofilm-Forming and Adhesive ST398 Human-Adapted MSSA Lineage Causing Septic Knee Arthritis Following Surgical Reconstruction

**DOI:** 10.3390/microorganisms9020305

**Published:** 2021-02-02

**Authors:** Viviana Cafiso, Flavia Lo Verde, Alessandra Zega, Giuseppe Pigola, Roberto Rostagno, Silvio Borrè, Stefania Stefani

**Affiliations:** 1 Department of Biomedical and Biotechnological Sciences, University of Catania, 95123 Catania, Italy; flavia.loverde@hotmail.it (F.L.V.); alessandra.zega@libero.it (A.Z.); gpigola@gmail.com (G.P.); stefanis@unict.it (S.S.); 2 Infectious Diseases Department of Sant’Andrea Hospital Vercelli, 13100 Vercelli, Italy; infettivi.vercelli@aslvc.piemonte.it (R.R.); silvio.borre@aslvc.piemonte.it (S.B.)

**Keywords:** biofilm, adhesion, ST398, *S. aureus*, genomics

## Abstract

Methicillin-susceptible (MSSA) and methicillin-resistant *Staphylococcus aureus* (MRSA) is a pathogen commonly found in bone and joint infections, including septic arthritis. *S. aureus* virulence and the frailty of affected patients can cause several complications; a prompt and specific antibiotic treatment can positively affect the outcome of patients. We carried out an in-depth genomic characterization by Illumina whole genome sequencing and bioinformatics of two biofilm-producing M1 and M2 ST398 MSSA causing septic knee arthritis not-responding to antimicrobial therapy. The strains were characterized for antibiotic resistance, biofilm and adhesive properties as well as genomics, single nucleotide polymorphism phylogeny, resistomics and virulomics. Our results showed that M1 and M2 MSSA were ST398-t1451-*agr*I-Cap5, susceptible to cefoxitin and resistant to erythromycin and clindamycin, traits consistent with the lack of the SCC*mec*-locus and the presence of the sole *bla*Z and *erm*T. Furthermore, M1 and M2 were biofilm-producing and largely potentially adhesive strains, as indicated by the adhesion gene profile. Our data characterized a new human-adapted ST398 MSSA lineage, representing a “fusion” between the human-animal independent ST398 and the Livestock Associated (LA) ST398 lineages, forming biofilm and genomically predicted high adhesive, characterized by different genomic adaptation conferring a great ability to adhere to the host’s extracellular matrix causing septic knee arthritis.

## 1. Introduction

Invasive *Staphylococcus aureus* can cause severe and chronic septic arthritis associated with a high rate of relapse [1], in part explained by its ability to produce biofilm and small colony variants (SCVs) [2]. Biofilms are sessile microbial communities embedded in an extracellular polymeric slime (EPS) matrix composed of polysaccharide, protein or external DNA (eDNA) living on surfaces [3,4].

Bacterial communities in biofilms alter some of their phenotypic (susceptibility to disinfectants and antimicrobial agents) and genotypic features (gene expression and protein production profiling) [5]. A biofilm’s life cycle has various steps: (1) a reversible attachment to a surface by van der Waals forces, steric interactions, an electrostatic (double layer) interaction and irreversible adhesion mediated by hydrophobic and hydrophilic interactions between bacteria and surfaces; (2) bacterial adhesion to the host matrix via microbial surface components recognizing adhesive matrix molecules (MSCRAMMs) and secretable expanded repertoire adhesive molecules (SERAMs); (3) production of microcolonies, with bacteria attaching to each other and producing additional polymeric substances; (4) microcolony maturation and mature biofilm formation with channels that enable nutrients to flow into the interior of the biofilm [6].

In recent decades, the clonal complex 398 (CC398) methicillin-resistant SA (MRSA) spread in animals and humans worldwide [7]. Human infections caused by CC398 MRSA were detected in pig farmers first in Europe and then in North America and Asia [7,8,9]. Two CC398 SA subpopulations associated both to serious infections —bloodstream infection, endocarditis, osteomyelitis, and necrotizing pneumonia—or mild infections—skin and soft-tissue infections—were reported. In details, the two CC398 subpopulations included a human-adapted CC398 SA clade characterized by the integrase group 3 prophage (ϕSa3), the immune evasion gene cluster (IEC) and the erythromycin-resistant gene *erm*(T), and a livestock-associated (LA) CC398 SA clade with the SCC*mec* locus (methicillin-resistance), the tetracycline-resistant gene *tet*M and carrying the ϕ2, ϕ6, or ϕAvb phages [10,11,12]. Moreover, recently human animal-independent ST398 MSSA were documented in several countries [13,14,15]. In our work, two strains of methicillin-susceptible *S. aureus* (MSSA) isolated before and during different antibiotic treatments came to our attention. The strains were related to a devastating infection sustained by MSSA.

The present work is the full characterization of the two MSSA strains, immediately confirmed as biofilm producers of the ST398 lineage.

## 2. Materials and Methods

### 2.1. Clinical Case

A 25-year-old athletic man, admitted to a private clinic with a diagnosis of contusive right knee trauma with complete rupture of the proximal middle third anterior cruciate ligament, underwent surgical ligament reconstruction with autologous patellar tendon, medial meniscectomy after chemoprophylaxis with 2 g of cefazolin.

Then, 11 days after surgery, the patient presented with intense pain associated with fever and evidence of edema and erythema of the medial face of the thigh, knee, and right leg. Arthrocentesis revealed corpuscular fluid positive for MSSA (M1 strain); consequently, the patient was treated with teicoplanin 800 mg and rifampicin 600 mg. Despite the antimicrobial therapy, the fever and inflammation persisted, hence the switch to 850 mg daptomycin + 750 mg levofloxacin + 4.5 g × 4 days tazobactam-piperacillin and a second surgery after 4 days—revealing the presence of fibrinoid tissue incorporating the neoligament, the screws of both the tibial and femoral graft and the meniscal root button—with all new cultures positive for *S. aureus*.

Next, 5 days after the second surgery, the persistence of inflammation, pain and fever, together with repeated isolation of MSSA from joint drainages (M2 strain)—even though in the presence of negative blood cultures—determined a new change in the antimicrobial therapy: first to daptomycin 850 mg and linezolid 600 mg × 2 days, and then to linezolid suspension. Nevertheless, the patient remained febrile and with persistent knee inflammation. Finally, 25 days after the second surgery, the new arthrocentesis revealed negative cultures, determining a new change in therapy, with discontinuation of daptomycin and initiation of dalbavancin infusions. The patient repeatedly underwent arthroscopic arthrolysis with biopsy samples negative for cultures.

**Figure 1:**
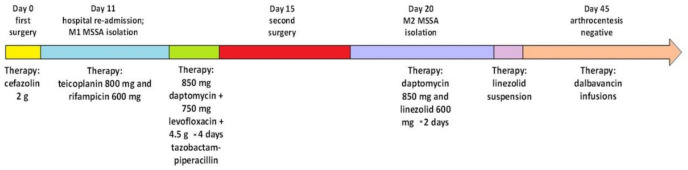
Timeline of M1 and M2 methicillin-susceptible (MSSA) isolation related to surgeries and treatment regimens.

A timeline of M1 and M2 MSSA isolation in relation to the surgeries and treatment regimens is shown in Figure 1.

### 2.2. Antibiotype

M1 and M2 MSSA antibiotypes were determined using minimum inhibitory concentration (MIC) determinations versus a panel of anti-Gram-positive antimicrobials, i.e., cefoxitin, vancomycin, teicoplanin, daptomycin, gentamicin, ciprofloxacin, cotrimoxazole, tetracycline, mupirocin, linezolid, dalbavancin, erythromycin, clindamycin, and rifampicin, according to the European Committee on Antimicrobial Susceptibility Testing guidelines version 10.0 (2020).

### 2.3. Biofilm and Slime Production

Biofilm and slime production were evaluated as previously published [16,17]. Briefly, a qualitative slime production assay was carried out in Congo Red Agar (CRA) incubating plates at 37 °C for 24 h and then at room temperature for 24 h. Black colonies were considered a positive slime-producer, whilst red colonies were considered to be a negative slime-producer [16]. The spectrophotometric assay was performed in microtitre plates and both isolates were grown in Tryptic Soy Broth (TSB) + 0.25% glucose. The reported value is the average of 12 measurements at 490 nm [17].

### 2.4. Haemolytic Ability

Haemolytic ability was investigated as previously published [18]. Briefly, *S. aureus* was cultured on blood agar plates (BioMérieux, Shanghai, China) as well as on homemade sheep blood agar plates prepared with Columbia blood agar powder (Oxoid, UK.). The bacteria were incubated at 35 °C in 5% CO_2_ (*v*/*v*) for 24 h and then underwent serial passages. The haemolytic properties were then investigated. Positive control strains and *S. aureus* ATCC25923, having a complete hemolytic phenotype and therefore to be considered as a positive control strain, were also used for comparative analysis.

### 2.5. Whole Genome Sequencing (WGS)

Whole genome sequencing (WGS) was performed with an Illumina Mi-Seq sequencing system using paired-end (PE) read libraries prepared by Nextera XT DNA Library Prep Kit (Illumina, San Diego, CA, USA) following the manufacturer’s protocol and whose quality was evaluated as previously published [19]. Raw reads were processed using FastQC (v.0.11.7) to assess data quality. The Cutadapter tool (v.1.16) implemented in Python (v.3.5.2) was used to remove residual PCR primers and to filter low-quality bases (Q_score < 30) and short reads (<150 bp) [20]. The filtered trimmed reads were included in the downstream analysis.

### 2.6. De Novo Genome Assembly

The de novo genome assembly was performed using SPAdes software (v3.12.0), producing a contig file for each sample. Post-assembly controls and metrics were generated using Quast (v.4.6.3). The CG viewer server was used to generate circular maps of the M1/M2 genomes (http://cgview.ca/).

### 2.7. Gene Annotation

The assembled contigs were processed by Rapid Annotations using Subsystems Technology (RAST) for microbial genome annotation (http://RAST.nmpdr.org).

### 2.8. Genomic Data Accession Number

The genomic reads were deposited in the Sequence Read Archive (SRA) of the National Center for Biotechnology Information (NCBI) Genome database under study accession n° SAMN16688054 and SAMN16688055 (BioProject: PRJNA675099).

### 2.9. Whole Genome Single Nucleotide Polymorphisms (wgSNPs)

SNP calls were carried out from the PE library raw reads as previously published [20]. The CSI Phylogeny tool (v1.4) [21] was used to identify the closest relationships between the strains and the different *S. aureus* Reference Genomes (RefGen) deposited in GenBank. The LA ST398 MRSA S0385 (acc. n° AM990992.1) and human animal-independent ST398 MSSA NM01 (acc. n° CP003045.1) were selected as Reference Genomes for SNP mapping in agreement with the phylogenetic results.

### 2.10. Whole Genome Single Nucleotide Polymorphisms Effect Prediction

The wgSNP effects of both mappings were evaluated by SnpEff (v.4.3T). High (HI), Low (LI), Moderate (MI), and ModiFier Impacting (MFI) effects were assigned according to the criteria previously published and in use in the tool. High impact: the variant is assumed to have disruptive impact in the protein, probably causing protein truncation and loss of function or triggering nonsense mediated decay. Low impact: the variant is assumed to be mostly harmless or unlikely to change the protein behavior. Moderate impact: the variant is a nondisruptive variant that might change protein effectiveness. Modifier impact: the variant is a usually noncoding variant or a variant affecting noncoding genes where predictions are difficult or there is no evidence of impact [22].

### 2.11. Genomic Epidemiology

Whole Genome Sequencing raw data were analyzed to investigate the genomic epidemiology with ResFinder (v.3.2) and Point Finder (v.3.1.0) services for the detection of acquired antimicrobial resistance (AMR) genes and to detect the known nsSNPs related to AMR genes [23]. Multi Locus Sequence Typing (MLST) was genomically checked using the MLST (v2.0) [24], spa-typing was confirmed by spaTyper (v.1.0) [25], the detection of acquired virulence genes was performed by VirulenceFinder (v.2.0) [26], the plasmid strain profile was investigated by the PlasmidFinder server (v.2.1) [27], mobile genetic elements (MGEs) were investigated by the Mobile element Finder (v1.0.3) [28], and prophages detection was performed by PHAge Search Tool (PHAST) [29]. Only the prophage region detected with a completeness score >90 was considered.

### 2.12. Core Genome Single Nucleotide Polymorphisms

The core genome SNP (cgSNP) detection shared by all strains was computationally carried out by Nullarbor on the LA ST398 MRSA S0385 (acc. n° AM990992.1) and human animal-independent ST398 MSSA NM01 (acc. n° CP003045.1) Reference Genomes.

## 3. Results

### 3.1. Phenotypic Description of the Strains (Antibiotype Biofilm Haemolytic Properties)

M1 and M2 MSSA strains showed a nearly complete susceptibility profile, being susceptible to cefoxitin, vancomycin, teicoplanin, daptomycin, gentamicin, ciprofloxacin, cotrimoxazole, tetracycline, mupirocin, linezolid, dalbavancin, and rifampicin; resistant only to erythromycin and clindamycin (Table 1).

**Figure 2:**
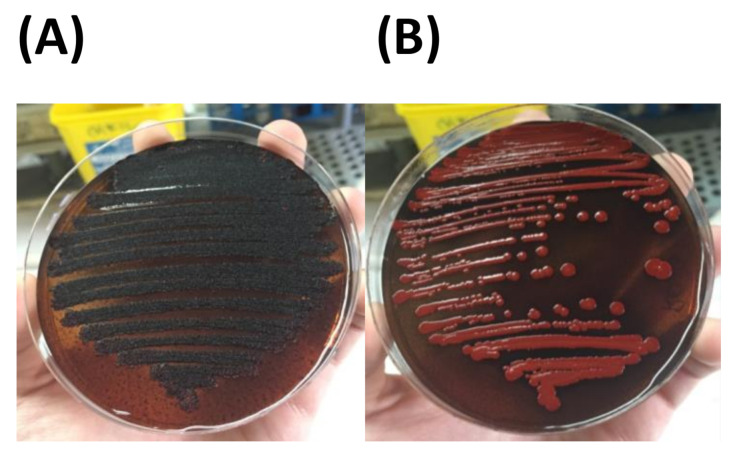
M1/M2 ST398 MSSA strains slime production (**A**), control *S. aureus* strain with negative slime production (**B**).

M1 and M2 MSSA, as immediately confirmed, were positive for the production of the extracellular polymeric slime (EPS) matrix formation in Congo Red Agar (Figure 2) and biofilm-producing in biofilm assays (O.D._490_ 0.4). Both strains were also not haemolytic on blood agar plates (Table 1).

### 3.2. Genomics

The de novo assembly of the M1 and M2 genomes yielded 57/92 contigs with a total length of 2,700,365/2,704,459 bp and a G+C content of 32.84/32.83%. The largest contig size was 412,322 bp for M1 and 405,071 bp for M2; the N50/N75 contig size was 190,663/80,862 for the first and 110,557/44,428 for the second one, with 98% genome coverage for both.

**Figure 3:**
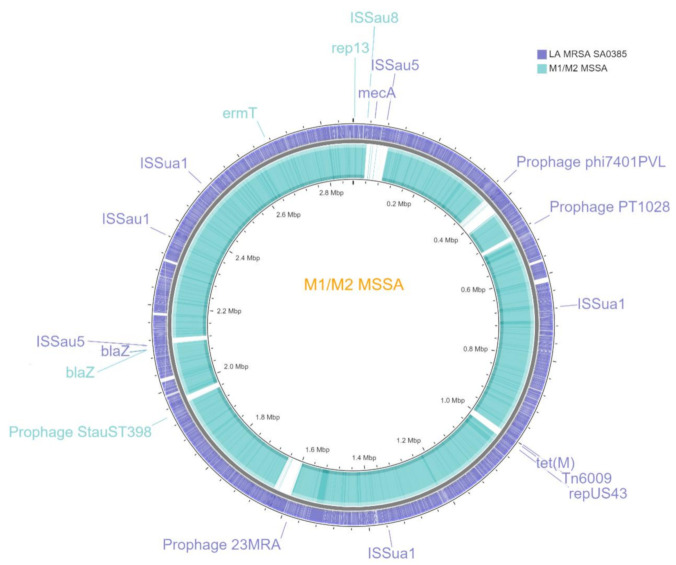
Circular maps of the comparison of M1 and M2 ST398 MSSA genomes versus the ST398 MRSA S0385 RefGen.

A circular graphic representation of the M1 and M2 MSSA genomes is provided in Figure 3.

**Figure 4:**
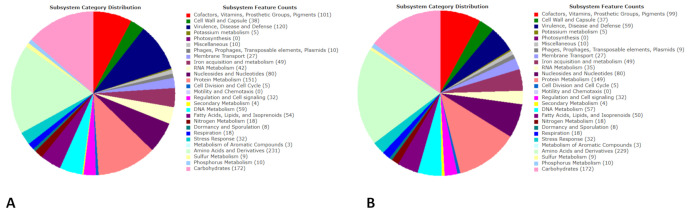
RAST annotation of predicted gene distribution in the M1 (**A**) and M2 (**B**) ST398 MSSA genomes.

The RAST annotation showed that the genomes had 2544/2708 coding sequences with a length of 2,701,787/2,740,680 bp and a G+C content of 32.8/32.9%. A total of 1206/1288 predicted genes were annotated according to the Subsystem Category Distribution performed by RAST, namely “Amino Acids and Derivatives” (*n* = 229/231), “Carbohydrates” (*n* = 172), “Cofactors, Vitamins, Prosthetic Groups, Pigments” (*n* = 99/101), “Cell Wall and Capsule” (*n* = 37/38), “DNA metabolism” (*n* = 57/59), “Fatty Acids, Lipids, and Isoprenoids” (*n* = 50/54), “Nucleosides and Nucleotides” (*n* = 80), “Protein Metabolism” (*n* = 149/151), “Stress Response” (*n* = 32), and “Virulence, Disease and Defense” (*n* = 59/120) (Figure 3). Several interesting subcategories were “Resistance to antibiotics and toxic compounds” (*n* = 23) and “Adhesion” (*n* = 23/84) in the “Virulence, Disease and Defense” category; “Osmotic stress” (*n* = 12) and “Oxidative stress” (*n* = 13) in the “Stress Response” category; “Protein biosynthesis” (*n* = 125/126) in the “Protein Metabolism” category (Figure 4).

### 3.3. Phylogenomics—M1/M2 ST398 MSSA Comparative Genomics—wgSNPomes

**Figure 5:**
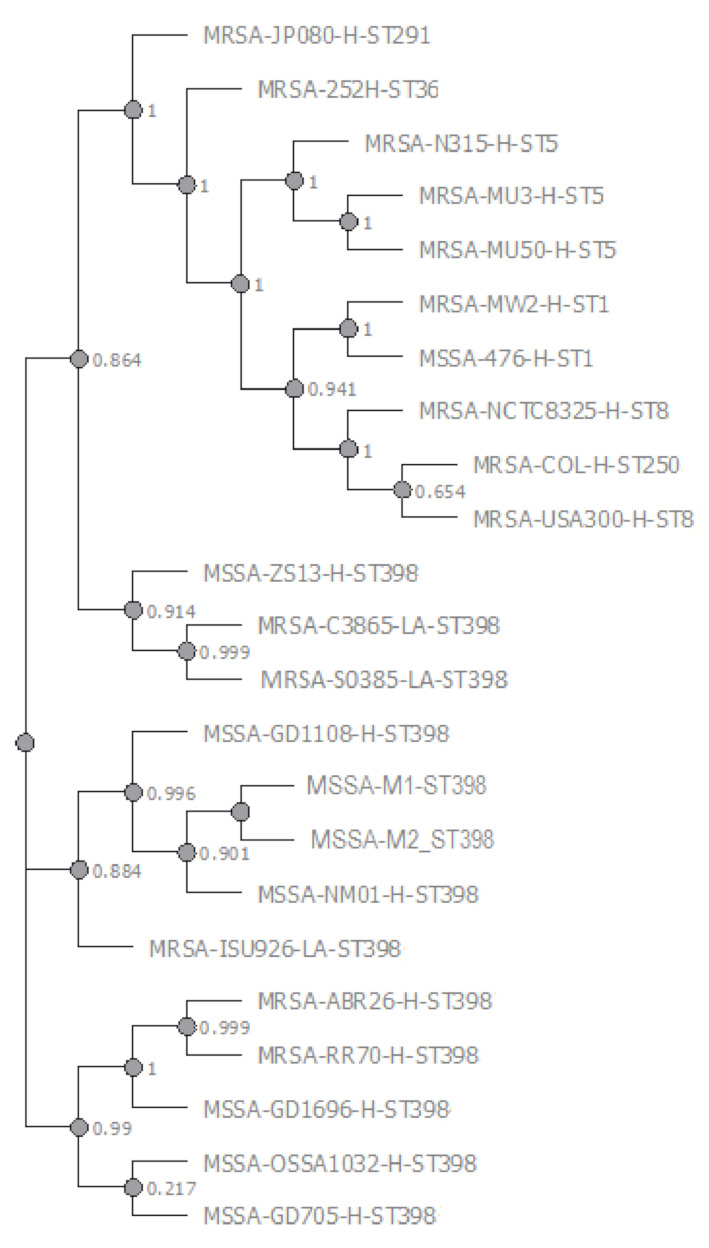
Phylogenetic Tree of M1 and M2 MSSA and other *S. aureus* strains referred to RefGen LA ST398 MRSA S0385.

Genomic epidemiology defined the M1 and M2 MSSA typing profile as ST398-t1451-*agr*I-Cap5. CSI wgSNP phylogeny distance analysis found the closest genomic similarity with the human animal-independent ST398 MSSA NM01 RefGen and other investigated human ST398 MSSA and MRSA genomes, followed by the LA ST398 MRSA RefGen as shown in the phylogenetic tree (Figure 5).

Comparative genomics of M1 and M2 ST398 MSSA—versus LA ST398 MRSA S0385 and human animal-independent ST398 MSSA NM01—highlighted characterizing human traits: (i) the lack of SCC*mec* cassette and therefore *mec*A/C genes; (ii) the plasmid *rep* gene 13; (iii) resistomes including *bla*Z and *erm*T; and unique traits that is no-shared features with the two reference ST398 strains: (i) the presence of a single copy of the MGE ISSau8; (ii) the presence of an intact copy of the Staphylococcus phage StauST398-4, Immune Evasion Cluster (IEC) β-converting Sa3 prophage [30] (Table 2).

The core genome SNP mapping performed both on the LA ST398 MRSA S0385 and the human animal-independent ST398 MSSA NM01 Reference Genomes showed a cgSNP distance indicating a close phylogenetic relationship between the M1 and M2 MSSA strains—with only one cgSNP difference in both mapping—as well as among the same strains and the human ST398 MSSA NM01 first and with LA ST398 MRSA S0385 Reference Genomes second, with 265/264 and 852/851 cgSNP differences respectively.

On the contrary, whole genome SNP mapping performed on both the LA ST398 MRSA S0385 and the human animal-independent ST398 MSSA NM01 Reference Genomes evidenced the M1 and M2 ST398 MSSA strain-related diversity due to a different wgSNP content, as shown in Table 3 and Supplementary (Tables S1 and S2).

### 3.4. Resistomics

Resistomics showed that the acquired resistance gene core of the M1 and M2 ST398 MSSA included the chromosomal β-lactamase coding *blaZ*, the penicillinase repressor *blaI*, the beta-lactam sensor/signal transducer *bla*R1, the 23S rRNA methyltransferase *erm*(T) located in the chromosome integrated plasmid pUR3912, conferring β-lactam, macrolides/lincosamides and streptogramin B (MLSb) resistance. No mutations were found in *rpo*B (rifampin resistance) and *fusA* (fusidic acid resistance), and no known mutations were detected in 23S rDNA (Table 1).

### 3.5. Pathogenomics

M1 and M2 ST398 MSSA virulomes were shaped by a mosaic of different virulence factors as follows: (i) adherence virulence determinants, namely the cell surface binding elastin *ebp*, the sortase-anchored surface proteins *isd*A-G, the MSCRAMM surface adhesins, i.e., biofilm intercellular adhesin PIA produced by ica-operon, clumping factor *clf*A/B, fibrinogen binding *fnb*A/B and cell wall anchored collagen adhesion *cna*, MHC-II analogous protein *map*, and the serine-aspartate repeat containing proteins *sdr*CDE; (ii) toxin coding genes, namely the hemolysin *hlg*ABC, *hlb*, *hld*, and *hly/hla*; (iii) exoenzyme coding genes, i.e., the zinc metalloproteinase aureolysin *aur*, the cysteine protease *ssp*ABC, the lipase lip, and the sortase *str*B; (iv) host immune evasion coding genes, i.e., *spa* (protein A belongs to the MSCRAMMs family) and *sbi*, both binding the IgG Fc portion interfering with opsonophagocytosis, staphylococcal complement inhibitor *scn* included in the IEC and *chp* (the immuno-modulator for the chemotaxis inhibitory protein of *Staphylococcus aureus* CHIPS), both contained in the conserved 3′ end of β-hemolysin (*hlb*)-converting bacteriophages βC-φs (β-converting Sa3 prophage), *Cap5* biosynthesis genes (the capsular polysaccharide synthesis enzyme implicated in the antiphagocytosis), *ads*A (adenosine synthase) and *geh* (activation of innate immune cells); v) *ess*ABC, *esa*AB and *esx*A (type VII secretion system) (Table 1).

Furthermore, BLAST alignments of the main adhesion genes versus LA ST398 MRSA and human animal-independent ST398 MSSA NM01 RefGenomes showed that M1 and M2 ST398 MSSA had the same wild type (WT) *clf*A, *clf*B, *fnb*A, *fnb*B, *coa*, *sdrE* and *sdr*C gene sequence as the human animal-independent ST398 MSSA NM01 RefGenomes, whilst the wild type (WT) collagen binding adhesin *cna* and mutated *sdr*D as the LA ST398 MRSA S0385 (Table 2).

### 3.6. M1 and M2 Shared HI and MI Effect wgSNPs

HI and/or MI effect nsSNP hotspots (≥4 nsSNPs) shared by M1 and M2 were found—only on the LA ST398 MRSA S0385 mapping (Ancestor protype genome of ST398 *S. aureus*)—in MSCRAMM family adhesin coding genes, i.e., clumping factor *clf*A/B (HI and MI SNPs mapped in these genes reflect the presence of a no mutated gene sequence described—in the above results of BLAST alignments of main adhesion genes as in human ST398 MSSA and therefore referred to as “frameshifting to WT gene sequence SNPs”), fibronectin binding coding gene *fnb*B (HI SNPs frameshifting to the WT gene sequence as in human-animal independent ST398 MSSA), superantigenlike exotoxin coding *ssl*11 (HI SNPs), extracellular adherence protein coding *eap* (MAP MHC class II analogous protein) (HI nsSNPs), serine-aspartate repeat protein *sdr*D (MI nsSNPs frameshifting to the mutated gene sequence as in LA ST398 MRSA S0385), collagen adhesin gene *cna* (MI nsSNPs frameshifting to the WT gene as in LA ST398 MRSA), Enoyl-acp reductase *FabI*, and staphylocoagulase coding gene *coa* (MI nsSNPs frameshifting to the WT gene as in human animal-independent ST398 MSSA) as shown in Table S1 and Table 2. In addition, HI nsSNPs were recovered in genes implicated in the metabolism or pathway of different compounds: (i) carbohydrates (*dha*M) (frameshifting to the WT gene) and lactose/galactose (*gal*E); (ii) phosphate (*pst*S); (iii) amino acid biosynthesis-arginine (*argJ*), tyrosine (*tyr*A) (frameshifting to the WT gene) and L-lysine (*dap*E); (iv) ammonium (*nrg*A); (v) oligopeptide transport (*opp*F); (vi) peptodoglycan turnover, i.e., N-acetylmuramoyl-L-alanine amidase *Lyt*H and autolysin (*ami*C gene); (vii) DNA (SAPIG0346 sirtuin2 family protein); (viii) SAPIG1607 (competence protein ComGC) (Table S1). MI effect SNP hotspots (≥4 nsSNPs) were also found in a miscellaneous group of genes, namely SAPIG0395 Phage holin, N-acetylmuramoyl-L-alanine amidase *Lyt*H, SAPIG1385-SAPIG2023-SAPIG2208 transposases, hyperosmolarity resistance protein Ebh, penicillinase repressor *blaI*, beta-lactam sensor/signal transducer *bla*R1, SAPIG2021 YOLD-LIKE family protein, SAPIG2024 tyrosine-type recombinase/integrase, multidrug efflux transporter *Sep*A, SAPIG2434 *Ara*C family transcriptional regulator *Rsp*, and SAPIG1887 and SAPIG2022 hypothetical proteins as shown in Table S1.

The strain-specific HI and MI nsSNPs mapped on the LA ST398 MRSA S0385 RefGen are shown in Table S1, while the strain-specific HI and MI nsSNPs mapped on the human animal-independent ST398 MSSA NM01 RefGen are shown in Table S2.

## 4. Discussion

ST398 *S. aureus* has recently emerged worldwide and is a frequent source of human infections, being involved in serious infectious diseases such as bloodstream infections (BSI) [31,32], prosthetic joint infections (PJI) [33,34] and in diabetic foot osteomyelitis (DFO) [35], whereas ST398 MRSA is mainly associated with infections in humans and animals [36]. ST398 MRSA and ST398 MSSA belong to distinct lineages [37].

*S. aureus* can switch to different lifestyles moving along a “continuum of cell modalities”—from planktonic, to sessile (biofilm), to quasidormant lifestyles (SCVs)—the modes of growth that are key to the ability to establish chronic, persistent and relapsing infections [38] under different environmental conditions or stress [38]. An array of metabolic pathways and surface MSCRAMMs, acting as receptors for host adhesion proteins of the joint extracellular matrix or implanted medical devices [39,40,41], confer to *S. aureus* a high affinity for the synovia, allowing this microorganism to be one of the most common agents in joint infections.

Our investigations characterized two ST398 MSSA isolates, being M2 a genetic evolution of M1 MSSA emerging under antimicrobial treatments, with a sticky behavior due to their in vitro slime and biofilm production. The severity of the infection, as described in the clinical section, did not correspond to the antimicrobial resistance profile but was instead related to the biofilm production ability and to the presence of several genes related to these sticky properties. The comparison of core genome SNPomes showed the close phylogenetic relationship between M1 and M2 ST398 MSSA compared to the human animal-independent ST398 MSSA NM01. Comparative genomics and genomic epidemiology evidenced that M1 and M2 represent a new human-adapted ST398 MSSA lineage having predominantly human-animal independent ST398 traits and few features associated to LA ST398. In details, they have the spa-type t1541, the *agr*-I type and the capsular type 5 as LA indicators [42], and the lack of the SCC*mec* cassette, a lower average core genome variation rate versus the human animal-independent ST398 MSSA NM01 RefGen, the IEC β-converting Sa3 ST398-4 prophage, the plasmid replication gene *rep13*, resistome including only *blaZ* and *erm*(T), and the adhesion coding gene sequences as in the human animal-independent ST398 MSSA NM01 markers. The resistome characterized by the presence of the β-lactamase *blaZ* (β-lactam Resistance), and the 23S rRNA methyltransferase *erm*(T) (MLSb Resistance) associated with the *tet*M lack is consistent with β-lactams and MLSb use in human therapy and tetracycline in animal feeds. These genomic traits strongly support the similarity between M1 and M2 ST398 MSSA and the human animal-independent ST398 MSSA genomic background.

Pathogenomics indicated that the M1 and M2 ST398 MSSA virulomes were prevalently shaped by a mosaic of “human-adapted like” virulence factors constituted by a large repertoire of wild type MSCRAMM surface adhesins, biofilm associated PIA, MHC-II analogous protein MAP, and the serine-aspartate repeat containing SdrCDE, ClfA/B and FnbA/B [43]. These adhesins along with collagen binding CNA, the surface binding elastin Ebp, and the sortase-anchored surface proteins IsdA-G, can affect the binding to several proteins, namely osteopontin, collagen, bone sialoprotein, vitronectin, fibronectin, and fibrinogen, conditioning the initial colonization and the intracellular immuno-evasion strategy.

Moreover, *S. aureus* can persist intracellularly after internalization in cultured osteoblasts [44] and switch to sessile form, the biofilm, escaping immune system clearance and antimicrobial therapy. M1 and M2 ST398 MSSA were slime and biofilm producers, a fundamental condition playing a key role in synovial environment for the establishment of a genuine biofilm-associated infections as septic arthritis.

Regarding toxin coding genes, M1 and M2 ST398 MSSA had a very poor toxin gene content made up of hemolysin coding genes *hlg*ABC, *hlb*, *hld*, and *hly*/*hla*, similarly to the human-animal independent ST398 MSSA, even though they were not haemolytic strains in agar plates likely for a dysregulation in *hla* expression, and showed the lack of enterotoxins and phage-encoded toxins [45]. A previous study demonstrated that α-hemolysin was a significant mediator of virulence in septic arthritis [45]. Superantigen toxins can play an important role in septic arthritis mortality and morbidity [45]. Concerning the host immune evasion coding genes, M1 and M2 ST398 MSSA had numerous genes coding for proteins implicated in immune evasion, namely protein A and Sbi both binding the IgG Fc portion interfering with opsonophagocytosis, the staphylococcal complement inhibitor Scn, the immuno-modulator for the chemotaxis inhibitory protein CHIP, Cap*5* biosynthesis genes involved in antiphagocytosis. In septic arthritis, *S. aureus* can evade the clearance by the host immune system using different staphylococcal defense mechanisms. First, protein A binds the Fc portion of immunoglobulin G and presents the Fab fragment of the antibody to the external environment; as a consequence, the Fc segment cannot bind the complement or signal polymorphonuclear leukocytes interfering with staphylococcal opsonization and phagocytosis [45]. Capsular polysaccharide can also interfere with opsonization and phagocytosis [46,47,48,49]. Type 5 capsule, present in M1 and M2 ST398 MSSA, could be also involved in internalization in cultured osteoblasts, therefore contributing to intracellular survival [50].

In conclusion, we characterized a new human-adapted ST398 MSSA lineage, biofilm producing and virtually highly adhesive to the host-derived matrix, characterized to be a “fusion” between a prevalence of human-animal independent genomic traits and few LA ST398 genomic features. Our data confirm the high genomic adaptive plasticity of *S. aureus*, conferring to this microorganism a great adaptability to be a joint infection pathogen.

## Figures and Tables

**Table 1:** Molecular characterization, virulomes, resistomes, MIC values and resistance gene SNPs in M1 and M2 strains.

Strains	MICs (mg/L)														Biofilm	Slime Production	Virulome	Resistome		
	**DAP**	**VAN**	**TEC**	**GEN**	**ERY**	**CLI**	**FOX**	**TET**	**MUP**	**CIP**	**SXT**	**RIF**	**LZD**	**DAL**						
**M1**	0.5	0.75	1	0.25	>32	ind	4	0.5	0.25	0.75	0.016	4	4	0.125	Producer	Positive	*ebp*	*blaZ* (β-lactam-R) *ermT* (Macrolide/Lincosamide and Streptogramin B-R)		
																	*isdA-G*			
																	*clfA/B*			
																	*fnbA/B*			
																	*cna*			
																	*map*			
																	*sdrCDE*			
																	*hlgABC*			
																	*hlb*			
																	*hld*			
																	*hly/hla*			
																	*aur*			
																	*sspABC*			
																	*lip*			
																	*strB*			
																	*spa*			
																	*sbi*			
																	*scn*			
																	*chp*			
																	*adsA*			
																	*geh*			
																	*essABC*			
																	*esaAB*			
																	*esxA*			
**M2**	0.5	0.75	1	0.25	>32	ind	4	0.5	0.25	0.75	0.016	4	4	0.125	Producer	Positive	*ebp*	*blaZ* (β-lactam-R) *ermT* (Macrolide/Lincosamide and Streptogramin B-R)		
																	*isdA-G*			
																	*clfA/B*			
																	*fnbA/B*			
																	*cna*			
																	*map*			
																	*sdrCDE*			
																	*hlgABC*			
																	*hlb*			
																	*hld*			
																	*hly/hla*			
																	*aur*			
																	*sspABC*			
																	*lip*			
																	*strB*			
																	*spa*			
																	*sbi*			
																	*scn*			
																	*chp*			
																	*adsA*			
																	*geh*			
																	*essABC*			
																	*esaAB*			
																	*esxA*			

**Table 2:** Comparative Genomics among LA ST398 MRSA S0385, human animal-independent ST398 MSSA NM01 and M1/M2 ST398 MSSA.

Strain	*mec* Gene	MLST	*spa*-Type	Capsular Type	*agr*-Group	Plasmid *rep* Gene	MGEs	Prophage	Resistome	Adhesin Genes								
										*clfA*	*clfB*	*fnbA*	*fnbB*	*CoA*	*sdrE*	*sdrC*	*sdrD*	*cna*
**LA-MRSA SA0385**	*mec*A	ST398	t011	Cap5	I	repUS43 CDS12738 (DOp1)	ISSau5 (2 copies) ISSau1 (4 copies) *Tn*6009	φ7401PVL PT1028 23MRA	*mec*A *bla*Z *tet*M	truncated	truncated	WT	truncated	Δ81bp	absent	Δ174bp	3SNPs	WT
**Human-animal independent MSSA NM01**	*-*	ST398	t571	Cap5	I	rep13 (pSSP1)	CT Cn_30859-ISSau1 (4 copies)	φNM3 R8A2B	*bla*Z *erm*T	WT	WT	WT	WT	WT	WT	WT	Δ54bp	ΔBdomain
**M1/M2** **MSSA**	*-*	ST398	t1451	Cap5	I	rep13 (pC194)	ISSau8 (1 copy)	StauST398-4	*bla*Z *erm*T	WT	WT	WT	WT	WT	WT	WT	3SNPs	WT

Legend: IS = Insertion Sequence; CT = Composite Transposon; MGEs = Mobile Genetic Elements.

**Table 3:** M1 and M2 ST398 MSSA core genome and whole genome SNPomes.

		SNPs Content on LA ST398 MRSA RefGen Mapping (CP003045.1)		SNPs Content on Human-Animal Independent ST398 MSSA RefGen Mapping (AM990992.1)	
		M1	M2	M1	M2
**cgSNPomes**	**cgSNPs *vs* RefGen**	852	851	265	264
	**cgSNPs M1 *vs* M2**	1	1	1	1
**wgSNPomes**	**Variant Rate**	1318	1401	531	1890
	**Variant Number**	2178	2091	5106	1436
	**MISSENSE SNPs (%)**	45.846	44.88	58.3106	57.164
	**NONSENSE SNPs (%)**	2.116	1.365	2.914	4.874
	**SILENT SNPs (%)**	52.08	53.74	38.98	37.962
	**HI Eff SNPs (%)**	4.02	3.44	1.35	6.163
	**MI Eff SNPs (%)**	33.501	33.01	6.53	27.978
	**LI Eff SNPs (%)**	34.87	35.323	4.32	18.09

Legend: cgSNPome = core genome SNPome, wgSNPomev: whole genome SNPome, HI EFF = High impact effect; MI = moderate impact effect, LI Eff = Low impact effect.

## Data Availability

The data sets generated for this study can be found in this article/Supplementary Materials. WGS raw reads were deposited at Sequence Read Archive (SRA) under study accession n° SAMN16688054 and SAMN16688055 (BioProject: PRJNA675099).

## Supplementary Materials

The following are available online at https://www.mdpi.com/2076-2607/9/2/305/s1, Table S1: HI and MI nsSNPs mapped on the LA ST398 MRSA S0385 RefGen; Table S2: HI and MI nsSNPs mapped on the human animal-independent ST398 MSSA NM01 RefGen.
